# Severe Neutropenia Due to Carbimazole and the Importance of Early Recognition and Management

**DOI:** 10.7759/cureus.81867

**Published:** 2025-04-08

**Authors:** Anup Banerjee, Ahmed Jamil, Eazaz Ali Khan

**Affiliations:** 1 Acute Medicine Unit, Stepping Hill Hospital, Stockport NHS Foundation Trust, Manchester, GBR; 2 Department of Medicine for Older People (DMOP), Stepping Hill Hospital, Stockport NHS Foundation Trust, Manchester, GBR

**Keywords:** carbimazole-induced agranulocytosis, granulocyte colony-stimulating factor (g-csf), graves', neutropenic sepsis, radioactive iodine (rai) therapy

## Abstract

Agranulocytosis is a rare but potentially life-threatening adverse effect of antithyroid medications, particularly carbimazole. This case report presents a well-structured and clinically relevant discussion of a rare but serious adverse effect of carbimazole therapy in a patient with Graves' disease. The case highlights the importance of early recognition and prompt discontinuation of the drug, which are crucial to preventing severe complications. We present the case of a 51-year-old female patient who was recently diagnosed with Graves' disease and developed agranulocytosis, leading to neutropenic sepsis after initiating carbimazole therapy. She initially presented to the medical same-day emergency care (SDEC) unit five weeks earlier with complaints of palpitations for two months, accompanied by weight loss, heat intolerance, irritability, and excessive sweating. Her thyroid function tests revealed a thyroxine (T4) level of 61.1 pmol/L and a thyroid-stimulating hormone (TSH) level of <0.01 mIU/L. An electrocardiogram (ECG) showed sinus tachycardia, while a thyroid ultrasound indicated an enlarged thyroid gland consistent with thyroiditis, along with a single U2 nodule in the right lobe. She was prescribed carbimazole and propranolol and was referred for outpatient endocrinology follow-up. After 4-5 weeks of treatment, she developed a fever, sore throat, and fatigue. A routine blood test ordered by her general practitioner (GP) revealed severe neutropenia, consistent with agranulocytosis. Carbimazole was immediately discontinued, and she was treated with broad-spectrum antibiotics and granulocyte colony-stimulating factor (G-CSF), leading to a gradual recovery. Carbimazole-induced agranulocytosis typically occurs within the first few months of therapy. Early discontinuation of the drug is associated with a favorable prognosis. Alternative treatment options, including radioactive iodine therapy and thyroidectomy, should be considered for long-term disease management.

## Introduction

Agranulocytosis, also known as agranulosis or granulopenia, is a serious hematological condition characterized by a marked reduction in granulocytes, a subset of white blood cells essential for immune defense. This condition significantly impairs the body's ability to fight infections, leading to an increased risk of severe or life-threatening complications. Agranulocytosis can be either congenital or acquired, with drug-induced cases being among the most common causes.

Carbimazole, a prodrug of methimazole, is widely used in the management of Graves' disease and other forms of hyperthyroidism. While generally well-tolerated, it carries the risk of rare but serious adverse effects, including hepatotoxicity and agranulocytosis. The incidence of carbimazole-induced agranulocytosis is estimated to be between 0.3% and 0.6% [1], with the highest risk occurring within the first few months of treatment initiation. Due to its potentially fatal consequences, early recognition and prompt management of agranulocytosis in patients receiving carbimazole are crucial. This case report highlights a patient who developed agranulocytosis following carbimazole therapy, emphasizing the importance of vigilance, timely intervention, and alternative treatment strategies for hyperthyroidism.

## Case presentation

A 51-year-old female patient presented to the medical same-day emergency care (SDEC) department with a two-month history of palpitations, weight loss, heat intolerance, irritability, and excessive sweating. Thyroid function tests revealed a thyroxine (T4) level of 61.1 pmol/L and a thyroid-stimulating hormone (TSH) level of <0.01 mIU/L. An electrocardiogram (ECG) showed sinus tachycardia (HR: 109 bpm).

**Figure 1:**
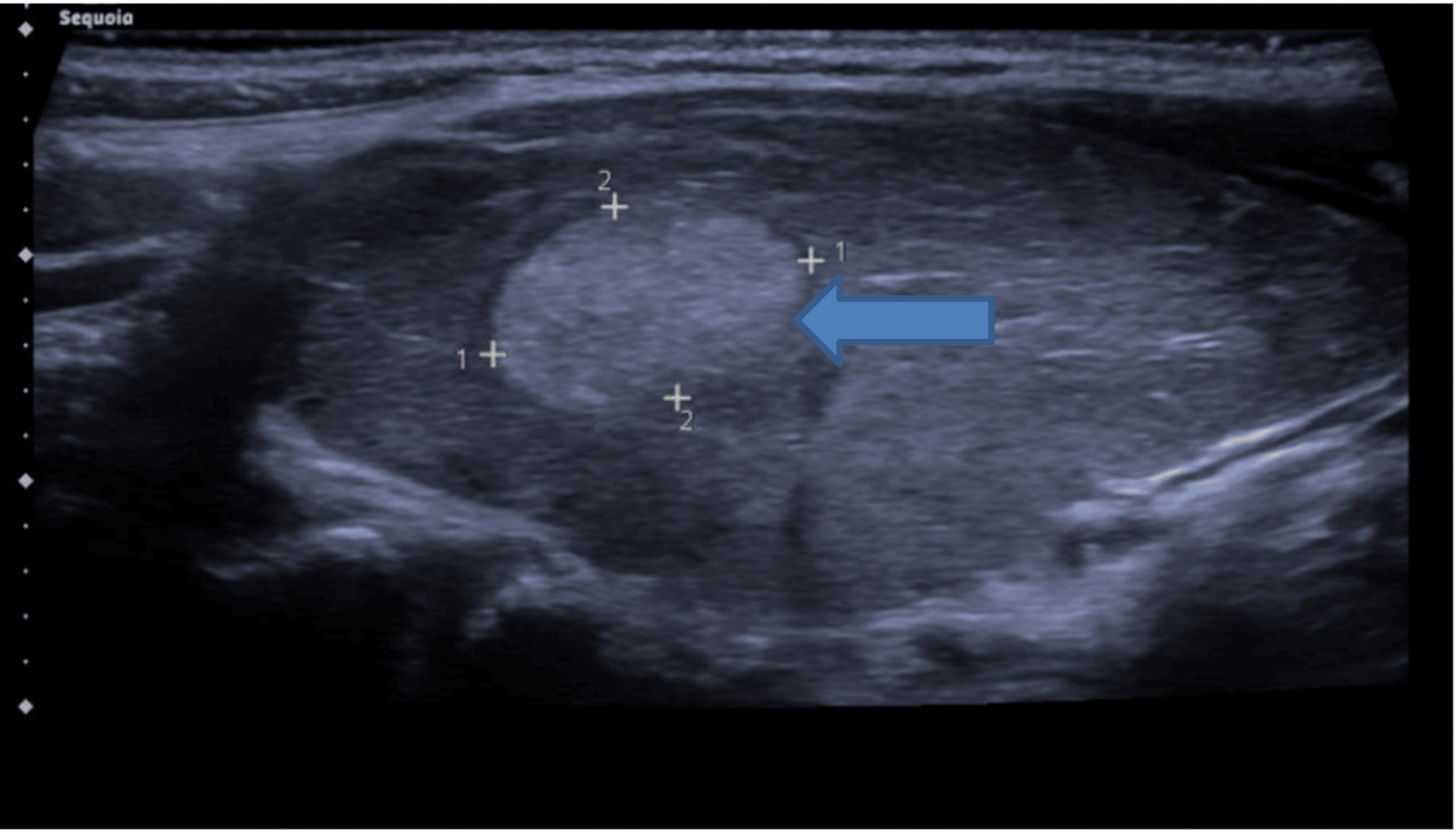
Ultrasound of the neck of the patient showing a single nodule

She was diagnosed with hyperthyroidism and started on carbimazole 40 mg daily and propranolol, with an outpatient endocrinology follow-up. A positive TSH receptor antibody (TSH-r Ab) test confirmed Graves' disease. Ultrasound (US) of the neck showed the thyroid gland is diffusely enlarged and appears hypoechoic with increased parenchymal vascularity, in keeping with thyroiditis. There is an ovoid, solid nodule within the right lobe that demonstrates a subtle spongiform texture and is surrounded by a hypoechoic halo. It would likely be isoechoic if compared to normal thyroid tissue and demonstrates no internal Doppler vascularity within (U2: benign). The nodule measures 15×9×12 mm. There was no retrosternal extension or tracheal deviation (Figure 1).

After five weeks of treatment, she developed a high fever, sore throat, and fatigue. Routine blood tests ordered by her general practitioner (GP) showed a white cell count (WCC) of 1.4×10⁹/L and a neutrophil count of 0.00×10⁹/L, prompting urgent hospital admission. On arrival, she was febrile (39°C), tachycardic, but hemodynamically stable. Repeat blood tests confirmed persistent agranulocytosis (WCC: 1.6×10⁹/L; neutrophils: 0.0×10⁹/L) and an elevated C-reactive protein (CRP) (96.6 mg/L; thyroid function tests: TSH: <0.01 mU/L and T4: 19.1 IU/L) (Table 1). 

**Table 1:** Laboratory results for the patient on admission showing the full blood count and thyroid hormones and antibody

Parameter	Patient value	Reference value
White cell count	1.6×10^9^/L	3.7-11.0×10^9^/L
Hemoglobin	109 g/L	115-165 g/L
Platelet count	287×10^9^/L	150-450×10^9^/L
Neutrophils	0.0x10^9^/L	1.7-7.5×10^9^/L
Serum free thyroxine	19.7 pmol/L	11.0-24.3 pmol/L
Serum thyroid-stimulating hormone	<0.01 mU/L	0.10-4.00 mU/L
Thyroid-stimulating hormone receptor antibody	19.1 IU/L	0.00-0.9 IU/L
Serum free triiodothyronine	10.6 pmol/L	3.5-6.5 pmol/L

Clinical examination revealed clear breath sounds on chest auscultation. Cardiac examination demonstrated normal heart sounds with no murmurs. Oropharyngeal inspection showed erythema without evident exudates. Chest radiography demonstrated no focal pulmonary or pleural abnormalities.

After a discussion with the hematology team, a diagnosis of carbimazole-induced neutropenia was made, carbimazole was discontinued, and the patient was started on granulocyte colony-stimulating factor (G-CSF). The endocrinology team advised against reintroducing antithyroid drugs (ATDs) and recommended radioactive iodine therapy or thyroidectomy for definitive treatment. The ear, nose, and throat (ENT) team deferred thyroidectomy during the acute phase due to surgical risks.

The patient was treated with intravenous broad-spectrum antibiotics (teicoplanin, metronidazole, and gentamicin) due to the risk of neutropenic sepsis. Blood cultures were negative, and after 48 hours of G-CSF therapy, her neutrophil count improved, and she became afebrile. She was discharged on oral antibiotics with endocrinology follow-up for the definitive management of her hyperthyroidism. Given the risk of cross-reactivity among thionamides, carbimazole or alternative ATDs were not reintroduced. Definitive therapy with radioactive iodine or thyroidectomy was planned in follow-up.

Prognosis

The patient's neutrophil count improved within two days, and she remained afebrile. She was discharged home on oral antibiotics (oral co-trimoxazole and clindamycin for five days) after discussing with microbiology. The patient is currently under endocrinology outpatient follow-up and awaiting radioactive iodine therapy for hyperthyroidism.

## Discussion

ATDs are widely utilized in the management of hyperthyroidism. However, these medications are associated with significant hematological adverse effects, ranging from mild leukopenia to severe complications such as agranulocytosis and, in rare cases, aplastic anemia [2]. Among these, agranulocytosis and hepatotoxicity represent the most serious and potentially life-threatening side effects of antithyroid therapy [2].

Carbimazole-induced agranulocytosis has an incidence of approximately 0.3-0.6% with a mortality rate of 21.5% [1,3]. Studies indicate that the risk of agranulocytosis is significantly increased in patients receiving methimazole doses ≥30 mg/day, particularly in individuals over the age of 40 [4].

Hyperthyroidism is a common endocrine disorder, with a higher prevalence in women (1-2%) compared to men (0.1-0.2%) [5]. While ATDs remain the first-line treatment, research suggests that carbimazole-induced agranulocytosis typically manifests within the first two to three months of therapy especially in patients receiving higher doses [6]. Given the serious nature of this complication, it is imperative for clinicians to educate patients on recognizing early warning signs, such as fever and sore throat, which may indicate neutropenia or agranulocytosis.

The precise pathophysiology of ATD-induced agranulocytosis remains incompletely understood, although several immunological mechanisms have been proposed. One hypothesis suggests that antibodies form against the drug when it binds to granulocyte cell membranes, leading to accelerated neutrophil destruction. Another theory proposes that antibodies target a drug-metabolite complex adsorbed onto neutrophil granulocytes, triggering autoimmune destruction. Additionally, ATDs may stimulate the production of autoantibodies, or an interaction between a granulocyte antigen and the drug may induce an immune response, resulting in neutropenia [7].

The clinical presentation of agranulocytosis varies, but the most commonly observed signs include ulcerative lesions of the upper respiratory tract mucosa, ulcerative-necrotic tonsillitis, gastrointestinal symptoms, fever, regional lymphadenopathy, and opportunistic infections such as fungal infections [8]. If a patient on ATDs develops these symptoms, immediate discontinuation of the drug is essential, along with a full blood count assessment to evaluate neutrophil levels. In cases where neutropenic sepsis is suspected, prompt initiation of broad-spectrum antibiotics is warranted, and the administration of G-CSF should be considered. G-CSF plays a crucial role in stimulating granulocyte proliferation and accelerating neutrophil recovery, thereby reducing the risk of severe infections and complications. Early recognition and intervention are essential for improving patient outcomes and preventing life-threatening consequences. Studies have demonstrated that treating ATD-induced agranulocytosis with G-CSF can reduce mortality rates from 21.5% to 5% [9,10].

The average recovery time in patients receiving G-CSF therapy is approximately 6.8 days. Following recovery from agranulocytosis, reintroducing an alternative ATD is not recommended due to the high likelihood of cross-reactivity among these medications [2,11]. For patients who have developed ATD-induced agranulocytosis, alternative treatment strategies such as radioactive iodine therapy or thyroidectomy are advised to prevent recurrence and provide long-term disease control.

## Conclusions

Antithyroid medications, including carbimazole, are commonly used to treat hyperthyroidism; however, agranulocytosis is a rare but potentially life-threatening side effect that may occur during the first few months of treatment. Close monitoring and regular follow-ups are crucial in this period to identify any adverse effects. If patients develop symptoms such as fever or a sore throat, blood tests should be performed immediately, and carbimazole should be discontinued if agranulocytosis is suspected.
